# 

**Published:** 1886-02

**Authors:** 


### CONTENTS.

### COMMUNICATIONS.

Dental Spiritualism........................................................ 57
Reminiscences ........................................................ 67
Gas Furnaces vs. Coal or Coke........................................................ 70

SELECTIONS.

Æsthetic Application of Dental Art........................................................ 72
Riggs's Disease........................................................ 78
Hydronaphthol—A New Antiseptic........................................................ 83
Local Treatment of Ranula........................................................ 89
Prevention of Bad Teeth........................................................ 90
Peculiar Effect of Corrosive Sublimate........................................................ 91
Hypodermic Medication........................................................ 92
Atropia in Acute Coryza........................................................ 93
Living with a Bullet in her Brain........................................................ 94
Items of Interest about the Pennsylvania Hospital........................................................ 95
Ninth International Medical Congress........................................................95–104
Menthol—A Local Anæsthetic........................................................ 96

SALMAGUNDI........................................................ 97

EDITORIAL.

Mississippi Valley Association of Dental Surgeons........................................................99–101
Association ........................................................ 105
Louisiana State Dental Society........................................................ 107

